# Implementation science research for the scale-up of evidence-based interventions for sickle cell disease in africa: a commentary

**DOI:** 10.1186/s12992-021-00671-x

**Published:** 2021-02-17

**Authors:** Joyce Gyamfi, Temitope Ojo, Juliet Iwelunmor, Gbenga Ogedegbe, Nessa Ryan, Amy Cohen, Obiageli Nnodu, Ambroise Wonkam, Charmaine Royal, Emmanuel Peprah

**Affiliations:** 1 Global Health Program and Department of Social and Behavioral Sciences, NYU School of Global Public Health, 14 East 4th Street, 3rd Fl, New York, NY 10003 USA; 2 Behavioral Science and Health Education, College for Public Health and Social Justice, Salus Center, Saint Louis University, 3545 Lafayette Avenue, Saint Louis, MO 63103 USA; 3 Department of Population Health, NYU School of Medicine, NYU Langone Health, 180 Madison Avenue,7th Fl, New York, NY 10016 USA; 4 Centre of Excellence for Sickle Cell Disease Research & Training (CESRTA), University of Abuja, Airport - Giri Expressway, Abuja, Federal Capital Territory Nigeria; 5 Division of Human Genetics, Faculty of Health Sciences - University of Cape Town, Anzio Road Observatory, Cape Town, 7925 South Africa; 6 Departments of African & African American Studies, Biology, Global Health, and Family Medicine & Community Health, Duke University, 1316 Campus Drive, 234 Ernestine Friedl Building, Box 90252, Durham, NC 27708 USA

**Keywords:** Global health, Implementation science research, Sickle cell disease, Africa, Scale-up

## Abstract

**Background:**

The burden of sickle cell disease (SCD) is greatest among African nations. Effective scalability of evidence-based interventions (e.g., newborn screening, health education, prophylaxis for infection, optimal nutrition and hydration, hydroxyurea therapy, blood transfusions, and transcranial Doppler (TCD) screening) is urgently needed particularly in these settings for disease management. However, Africa is constrained by limited resources and the lack of capacity to conduct implementation science research for proper understanding of context, and assessment of barriers and facilitators to the uptake and scalability of evidence-based interventions (EBI) for SCD management.

**Main Body:**

We outline implementation science approaches to embed EBI for SCD within the African context and highlight key implementation research programs for SCD management. Building implementation research capacity will meet the major need of developing effective life-long and accessible locally-tailored interventions for patients with SCD in Africa.

**Conclusion:**

This commentary communicates the importance of the application of implementation science methodology to scale-up evidence-based interventions for the management of SCD in order to reduce pain, prevent other morbidities and premature death experienced by people with SCD in Africa, and improve their overall quality of life.

## Background

Effective scalability of health interventions is urgently needed particularly in low-and middle- income countries (LMICs), considering the constraints these countries bear, and the lack of capacity to conduct implementation science research. For Africa, sickle cell disease (SCD), the inherited blood disease characterized by anemia, severe pain and other vasoocclusive complications, and early mortality, has significant financial, social, and psychosocial impacts and burdens individuals, families, and health systems. Currently it is projected that over 300,000 individuals are born annually with SCD in Africa [1, 2]. Comprehensive clinical care programs have reduced premature childhood deaths related to SCD in high-income countries including the USA where approximately 70% reduction in mortality is observed [3, 4]. In sharp contrast, it is estimated that 50–90% of children with SCD born in Africa die before the age of 10 [5]. The vast majority of people with SCD in Africa are not receiving evidenced-based health care (e.g., newborn screening, health education, prophylaxis for infection, optimal nutrition and hydration, hydroxyurea (HU) therapy, blood transfusions, and transcranial Doppler (TCD) screening), even though its use effectively reduces morbidity and mortality [6]. Cost and lack of knowledge are significant barriers to the use of evidenced-based interventions (EBIs) to address SCD in Africa [7]. Additionally, factors such as the acceptability, appropriateness, or feasibility of the interventions can also impede uptake. Implementation science strategies may be used to enhance the adoption and sustainability of clinical programs. While existing literature show that the systematic uptake of research findings and evidence-based practices is a desired outcome of implementation research, there is currently limited evidence of such interventions assessing sustainability and other implementation outcomes at the outset of implementation in Africa [8, 9]. Our team recently conducted a systematic review which synthesized reported implementation outcomes for the therapeutic use of HU for SCD management in resource-constrained settings including Africa. Of the 35 full-text articles reporting on empirical studies of HU for management of SCD that were conducted in LMICs, only two cross-sectional surveys (*n*=2) and one cohort study (*n*=1) reported implementation of HU for SCD management, namely regarding implementation outcomes of adoption, cost, and acceptability. These studies were conducted exclusively among pediatric and adults populations in clinical settings in Nigeria (*n*=2), and Jamaica (*n*=1). Findings suggest that adoption is low, as observed through reported provider practices and patient adherence, in part shaped by misinformation and fear of side effects among patients, provider beliefs regarding affordability and organizational challenges with procuring the medicine [9]. This commentary communicates the importance of the application of implementation science methodology to scale-up evidence-based interventions for the management of SCD in order to reduce pain, prevent other morbidities and premature death experienced by people with SCD in Africa, and improve their overall quality of life.

### Implementation science approach to embed EBIs for SCD in the African Context

Implementation science, defined as the scientific study of methods to promote the systematic uptake of research findings and other evidence-based practice into routine practice [10] guided by implementation frameworks (e.g., CFIR- Consolidated Framework for Implementation Research) has the potential to identify the factors, processes, and methods that can successfully embed EBIs for SCD into policy and practice [11]. Implementation outcome measures including *acceptability, adoption, appropriateness, costs, feasibility, fidelity, penetration, and sustainability* [12] are not well defined in the existing literature for SCD interventions in Africa. There is an urgent need, and an opportunity, to help improve SCD outcomes of individuals in Africa by building local capacity to utilize well-studied implementation frameworks (i.e., *PARIHS* (Promoting Action on Research Implementation in Health Services; EPIS (Exploration, Preparation, Implementation, Sustainment), etc.) [13, 14] and implementation strategies (i.e., practice facilitation via provision of external expertise on practice redesign, and a tailored approach to implementing guideline-concordant care) [15, 16], to enable the scale-up of the aforementioned EBIs. For example, currently, HU treatment programs for SCD management has been initiated in several African countries (e.g., Ghana, Tanzania, Democratic Republic of the Congo, and Nigeria) [6, 17, 18], and HU use shows great promise of significant reduction in the number of painful SCD episodes, acute chest syndrome, transfusions, and hospitalizations; however, these programs have not been systematic, nor have they included a thorough assessment of implementation research outcomes.

What is currently lacking within Africa is a comprehensive multicomponent implementation strategy that explores for example, the reach, uptake, and long-term sustainability of EBIs for SCD. This strategy, would ideally incorporate patient /provider/ community/ policy engagement [19] for the long-term management of SCD. A prospective cohort study conducted from 2006-2011 by Uyoga and colleagues (2019), which comprised children with SCD, living in Kilifi, Kenya, highlights the disproportionate morbidity and mortality in children born with SCD [20]. This longitudinal cohort study focused on clinical outcomes, with minimum stakeholder engagement (e.g., caretakers, community members, policymakers) in the research process. Effective stakeholder engagement promises to move the translation of clinical findings to context-specific implementation of EBIs to improve the health of SCD patients and result in population level impact. The implementation of EBIs for SCD require engagement of stakeholder groups that have historically been under-represented in implementation and dissemination research in many resource-constrained settings. Including multilevel stakeholders (patients, providers, organizations, and health systems) and at the policy level from the onset will garner viewpoints and invaluable information to ensure adequate treatment, successful program implementation, and scale-up [21–23].

### Key implementation research programs for SCD management

EBIs needed to manage SCD exist. The American Society of Hematology Sickle Cell Disease Guidelines provide SCD management recommendations including broad pain management approaches, pharmacological and nonpharmacological interventions and analgesic delivery [24] blood transfusion support [25], and prevention, diagnosis, and treatment of cerebrovascular disease in children and adults with SCD [26], which recognizes the challenges and opportunities that are unique to LMICs including cost-effective approaches. Further, dearth of SCD management resources, and limited number of physicians with expertise in hematology and implementation science research training are significant barriers to providing effective SCD management in many LMICs including Africa. The next opportunity lie in building capacity in the use of implementation science research methods for proper understanding of context, and assessment of barriers and facilitators. Implementation research programs should include improvement in newborn screening through community mobilization, adoption of HU, penicillin prophylaxis, folate supplementation, and patient/caretaker education for SCD management [27]. Additionally, SCD care and management should be well integrated into healthcare systems by aligning the program with the strategic aims/policies of the organization and incorporate training on task-shifting/ task sharing of SCD care to non-hematologists (i.e., primary care nurses), and undertake assessment of the effectiveness of the EBI, cost and its cost-effectiveness in that setting. Building implementation research capacity will meet the major need of developing cost-effective, life-long and accessible interventions for patients with SCD in Africa. The high prevalence of SCD in Africa provides opportunities for such implementation research efforts, that will require implementation studies engaging multilevel stakeholders, to understand environmental, social, and genetic risk factors associated with disease outcomes. Such efforts will enhance the reach, uptake, long-term sustainability and scale-up of EBIs to reduce pain, prevent other morbidities and premature death experienced by people with SCD in Africa, and improve their overall quality of life.

## Conclusion

This commentary highlights the need for EBIs for SCD management across Africa. It also outlines key implementation research programs that should employ implementation science strategies and capacity building activities for SCD management. We note the success of EBIs in high-income countries that have reduced childhood SCD-related deaths but yet resource-constrained settings, including Africa lack access to EBIs. We acknowledge the critical importance of investigators implementing clinical trials in Africa for SCD management to plan for sustainability at the onset and throughout the implementation process, considering the tremendous amount of resources required to implement these trials. We, therefore advocate for a comprehensive multicomponent implementation strategy with strong patient and community stakeholder engagement, and the need to build capacity at the provider and health systems level to conduct implementation science research for proper understanding of context, and assessment of barriers and facilitators to the uptake, sustainability, and scalability of EBIs for SCD management within the African context.

## Data Availability

Not applicable
